# Precision Targeting of NF-κB Signaling in Lupus Nephritis

**Published:** 2020-12-28

**Authors:** Dawn J. Caster, David W. Powell

**Affiliations:** Department of Medicine, Division of Nephrology and Hypertension, University of Louisville School of Medicine, Kentucky, USA

**Keywords:** Lupus nephritis, Systemic lupus erythematosus, Nuclear factor kappa B, TNIP1, ABIN1, Lupus

## Abstract

Lupus Nephritis (LN) is the leading cause of morbidity and mortality from Systemic Lupus Erythematosus (SLE) and enhanced activation of the transcription regulator nuclear factor kappa B (NF-κB) is implicated as a central player in the development and progression of LN. SLE and LN are proposed to develop through a “two-hit” process of genetic mutation or variants providing susceptibility to disease provoking molecular events in response to environmental triggers (viral infection, medication, etc.). Many of the susceptibility genes identified in association with LN are involved in NF-κB regulation and loss of function of some of the protein products in animal’s results in protection from or development of SLE and LN phenotypes. This short commentary will discuss these factors and implications in precision treatment of LN.

## ABOUT THE STUDY

NF-κB controls the expression of the cytokines/chemokines and other immune modulators that mediate the cellular and molecular events in the LN pathogenesis [1,2]. These events include loss of immune tolerance from impaired myeloid cell apoptosis and clearance and release of nuclear products, activation of auto-reactive T cells and subsequent B cell activation and production of autoantibodies and formation of circulating Immune Complexes (IC), and glomerular infiltration and activation of inflammatory cells (neutrophils, monocytes) following IC deposition [3–6]. Many of the environmental triggers for LN activate these cellular processes and NF-κB through toll-like receptors (TLR) [7].

Variants in several genes that express components in the TLR/NF-κB signaling axis were identified in association with LN, including TLR 3/7/9, MYD88, IRAK1, TNFAIP3, and TNIP1 [3]. Brady et al. recently reviewed the associate of TNIP1 polymorphisms with LN [8]. Of the five primary TNIP1 polymorphisms identified as risks for SLE, only two (rs7708392 and rs4958881) have been confirmed with association with LN. TNIP1 expresses ABIN1, a polyubiquitin binding protein that functions as a physiological inhibitor of NF-κB by binding to polyubiquitin moieties on upstream regulators and this process is also facilitated by ABIN1 coupling with A20, the product of TNFAIP3 [9]. Nanda et al. reported that transgenic mice expressing an ABIN1 mutation (ABIN1[D485N]) disrupting the NF-κB inhibitory function spontaneously and progressively develop a number of SLE-associated phenotypes, including splenomegaly and elevated circulating IC and pathogenic antinuclear antibody (ANA) and Anti-ds DNA autoantibodies [5]. This report also showed that TLR 1–9-stimulated NF-κB activation was enhanced in myeloid and B cells isolated from ABIN1[D485N] mice, as compared with cells from wild-type mice. Caster et al. reported that ABIN1 [D485N] mice also develop progressive glomerulonephritis (GN) with pathologic features of the most common and severe form of human LN (Class IV) [4]. Nanda et al. have also reported that MYD88 and TLR7 deficiency and inhibition of IRAK1 and IRAK4 kinase activity suppressed the development of autoimmunity and LN in ABIN1[D485N] mice [5,10,11]. Kuriakose et al. showed that ABIN1 deficient mice also spontaneously develop SLE-associated autoimmune phenotypes and progressive, proliferative immune complex-mediated GN and that MYD88 and TLR7/9 deficiency suppressed development of GN in these mice [6].

Collectively, these findings suggest that TNIP1 variants and resulting loss of ABIN1 molecular function contribute to development of SLE and LN due to uncontrolled regulation and subsequent enhanced NF-κB activation through the TLR7/9/MYD88/IRAK1/4 signaling axis. This suggests that pharmacologic inhibition of TLRs, MYD88, and IRAK1/4 could provide viable precision treatment options for LN in patients with rs7708392 and rs4958881 variants in TNIP1 or variants in other NF-κB regulatory genes. Another promising therapeutic direction for patients with these genotypes is use of monoclonal antibodies against NF-κB effector molecules that are implicated in LN, such as IFNs, IL-6, IL-17, IL-23, and IP-10. Several monoclonal antibodies targeting these cytokines are in development or approved for other indications. Anifrolumab, a human monoclonal antibody to type I interferon reduced SLE activity in patients with moderately to severe SLE and is currently being evaluated in LN (NCT02547922) [12]. IL-6 receptor inhibitors have been FDA approved for rheumatoid arthritis (sarilumab, tocilizumab). Sirukumab, an anti- IL-6 monoclonal antibody failed to show efficacy in LN, but the investigators comment that a small subset of subjects appeared to have response to therapy [13]. Multiple IL-17 and IL-23 inhibitors are used for the treatment of psoriasis (IL-17: ixekizumab, brodalumab, secukinumab; IL-23: guselkumab, risankizumab), but have not been evaluated in SLE and LN.

## CONCLUSION

The identification of SLE and LN patients who have enhanced NF-κB activation and have elevated levels of effector cytokines may predict which patients are most likely to benefit from these specific treatments.
